# Alkaloids Modulate Motility, Biofilm Formation and Antibiotic Susceptibility of Uropathogenic *Escherichia coli*

**DOI:** 10.1371/journal.pone.0112093

**Published:** 2014-11-12

**Authors:** Devendra H. Dusane, Zeinab Hosseinidoust, Bahareh Asadishad, Nathalie Tufenkji

**Affiliations:** Department of Chemical Engineering, McGill University, Montreal, Quebec, Canada; Ghent University, Belgium

## Abstract

Alkaloid-containing natural compounds have shown promise in the treatment of microbial infections. However, practical application of many of these compounds is pending a mechanistic understanding of their mode of action. We investigated the effect of two alkaloids, piperine (found in black pepper) and reserpine (found in Indian snakeroot), on the ability of the uropathogenic bacterium *Escherichia coli* CFT073 to colonize abiotic surfaces. Sub-inhibitory concentrations of both compounds (0.5 to 10 µg/mL) decreased bacterial swarming and swimming motilities and increased biofilm formation. qRT-PCR revealed a decrease in the expression of the flagellar gene (*fli*C) and motility genes (*mot*A and *mot*B) along with an increased expression of adhesin genes (*fim*A, *pap*A, *uvr*Y). Interestingly, piperine increased penetration of the antibiotics ciprofloxacin and azithromycin into *E. coli* CFT073 biofilms and consequently enhanced the ability of these antibiotics to disperse pre-established biofilms. The findings suggest that these alkaloids can potentially affect bacterial colonization by hampering bacterial motility and may aid in the treatment of infection by increasing antibiotic penetration in biofilms.

## Introduction

Urinary tract infections (UTIs) are the second most frequent bacterial infection worldwide and account for almost half of all hospital acquired infections [1]. Strains of *Escherichia coli* known as uropathogenic *E. coli* (UPEC) are the major causative agent of UTI in humans [2], [3]. These opportunistic intracellular pathogens can colonize both biotic (mucosal epithelial cells lining the urogenital tract) [4] and abiotic (indwelling medical devices such as catheters) surfaces [2], [5].

**Figure 1:**
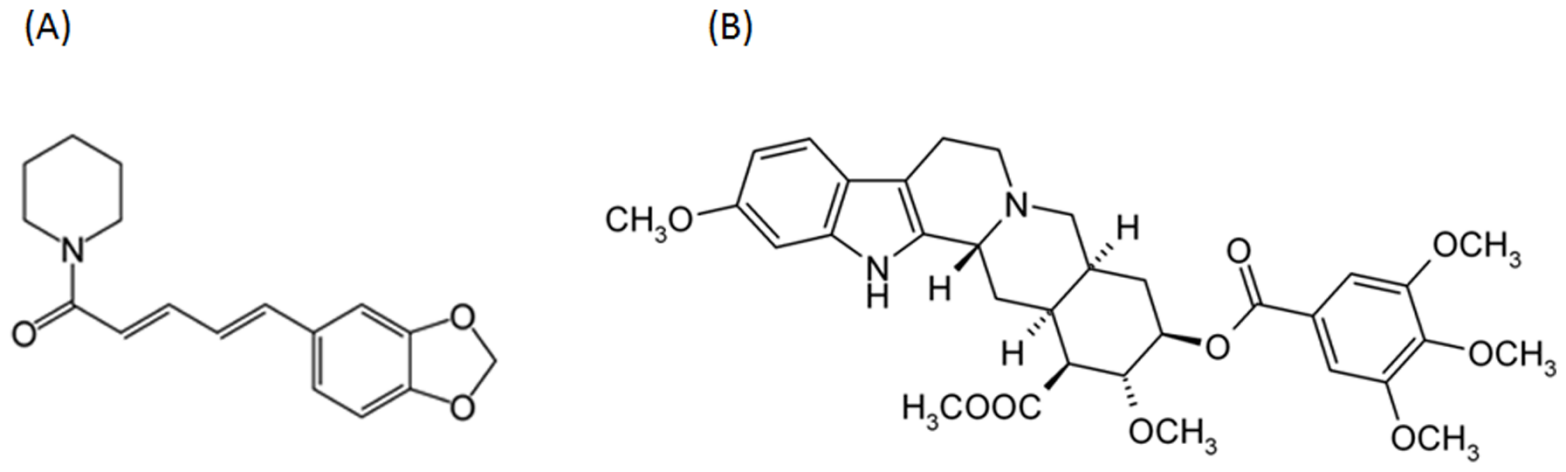
Molecular structure of alkaloids used in this study. (A) piperine, molecular weight: 285.34 and (B) reserpine, molecular weight: 608.68.

Natural compounds have long been considered for the treatment of UTIs, either alone or in combination with antibiotics. Certain plant-derived products have been shown to exhibit antimicrobial properties towards UTI-related pathogens [6]–[8] or to modulate bacterial virulence factors such as bacterial motility [9]–[11]. Of potential interest to treatment of UTIs are the alkaloids piperine (PIP) and reserpine (RES). Alkaloids are a group of naturally occurring chemical compounds known as secondary metabolites, found mainly in various genera of seed plants [12]. PIP is found in *Piper nigrum* or black pepper and RES is found in the dried roots of *Rauwolfia serpentina* (Indian snakeroot). Chemical structures of RES and PIP are presented in Figure 1. Both compounds are believed to have diuretic properties and have been used to treat kidney diseases for centuries [13], [14]. There are reports in the literature supporting the hypothesis that these compounds can be used for the treatment of UTIs [15]. However, it is known that these compounds are not strongly bactericidal and their mode of action remains unknown.

Previous reports from our laboratory suggest that certain natural compounds could affect bacterial colonization by affecting bacterial motility and biofilm formation [9]–[11]. Bacterial colonization of biotic or abiotic surfaces results from two distinct physiological processes, namely bacterial adhesion and biofilm formation [16]. Biofilms usually form after bacterial adhesion, however, not all single bacterial cells adhering reversibly or irreversibly engage into a biofilm mode of growth. Biofilms are structured, surface-associated microbial communities, embedded in a self-produced matrix of extracellular polymeric substances (EPS) [17], [18]. Bacteria growing in biofilms are generally very persistent, requiring high doses of antibiotics for treatment [19], [20]. The EPS matrix can limit oxygen availability and reduce bacterial metabolic activity, which is an important factor protecting biofilm bacteria from antibiotics [21]. The EPS matrix also introduces a diffusion limitation for drugs [22], [23], thus most of the antibiotics cannot penetrate to the full depth of the biofilm, resulting in reduced rates of killing of bacterial cells present within the biofilm [22]–[24].

Bacterial cell surface appendages (*i.e.*, fimbriae and flagella) play a major role in biofilm formation [25], [26]. Fimbriae are hair-like surface appendages that are directly involved in the attachment of bacteria to various surfaces [25]–[27]. Flagella are helical filaments that protrude from the cell body and are largely responsible for bacterial motility. A mutation in, or down-regulation of major UPEC flagellar genes could impede or completely block bacterial motility [28], [29], leading to an increase in biofilms [30]–[32], but also decreasing the ability of bacteria to disseminate and cause infection [3].

In the present study, we investigated the hypothesis that exposure to the alkaloids PIP and RES could affect the colonization behaviour of the uropathogenic bacterium *E. coli* CFT073. We examined bacterial motility, ability to form biofilms and expression of genes relevant to bacterial motility and surface attachment in the presence of these alkaloids. Finally, we examined the effect of PIP and RES on the ability of the antibiotics ciprofloxacin and azithromycin to penetrate into and disperse pre-established *E. coli* CFT073 biofilms.

## Materials and Methods

### Bacterial strains and chemical agents

*Escherichia coli* strains CFT073, CFT073 P*_fliC_-lux* and CFT073 Δ*fliC* were used in this study (Table S1). Bacterial cultures were grown in lysogeny broth (LB) and, unless otherwise stated, ampicillin (100 µg/mL) and kanamycin (50 µg/mL) were added to cultures of *E. coli* CFT073 P*_fliC_-lux* and *E. coli* CFT073 Δ*fliC*, respectively. PIP and RES were obtained from Sigma (Canada) and stored at 4°C as 1 mg/mL stock solutions in methanol.

### Bacterial growth

Wild type and mutant (Δ*fli*C) *E. coli* CFT073 strains were cultured in the presence or absence of PIP and RES (at concentrations 0.5, 5, 10 and 50 µg/mL). Overnight cultures, grown in LB at 37°C with shaking at 150 rpm, were diluted 1000-fold with LB medium. The cell suspension, containing 1×10^6^ cells/mL was distributed into sterile 96-well polystyrene microtiter plates (BD Falcon, USA) and incubated at 37°C. Possible effect of methanol in the alkaloid stock solution on bacterial growth was accounted for by adding the equivalent methanol concentrations to the control wells. The optical density of the bacterial culture (OD_600_) was recorded at 30 min intervals for 24 h using a Tecan Infinite M200 Pro plate reader (Tecan, Switzerland). All experiments were performed in triplicate.

### Bacterial motility

Swimming assays were performed on soft LB-agar plates containing 0.25% agar with PIP or RES (final concentrations of 0.5 and 5.0 µg/mL) [33]. Swarm plates were prepared by adding 0.5% Eiken agar (Eiken Chemical, Japan) to LB medium, supplemented with 0.5% glucose [29] and PIP or RES (0.5 and 5.0 µg/mL). Control plates without PIP or RES were also prepared and all plates were allowed to dry overnight at room temperature before use. An overnight culture of *E. coli* CFT073 (wild type and Δ*fliC* mutant) was diluted 1000-fold in LB and incubated at 37°C until early stationary phase (OD_600_≈0.5). Swarm plates were spot-inoculated on the agar surface with a 5 µL drop of bacterial culture. Swimming plates were seeded below the agar surface using a sterile inoculating needle. The plates were incubated at 37°C for 24 h, after which the diameters of swimming and swarming zones were measured and recorded [29]. The motility assays were performed in triplicate plates.

### Expression of flagellar fliC gene

*E. coli* CFT073 P*_fliC_*-*lux* was grown in LB overnight (37°C, 150 rpm). This culture was diluted 1000-fold and aliquots (containing 10^6^ cells/mL) were mixed with PIP and RES (0.5 and 5.0 µg/mL). The cultures were incubated at 37°C in a 96 well microtiter plate (clear bottom, white polystyrene). Growth (OD_600_) and luminescence were recorded every 15 min for 4 h using a plate reader. Expression of the flagellar gene, *fli*C, was quantified by normalizing the measured luminescence to the cell concentration: luminescence/(OD_600_–OD_600, initial_). Potential interference of PIP or RES with luminescence was verified using the Steady-Glo luciferase assay (Promega, Canada). PIP and RES (0.5, 5 and 50 µg/mL) were added to the luciferase assay mixture in 96 well microtiter plates (as per the manufacturer's instructions) and luminescence was recorded every 15 min for 5 h.

### Biofilm assays

*E. coli* CFT073 (wild type and Δ*fli*C mutant) were grown in LB overnight (37°C, 150 rpm). The culture was diluted 1000-fold in LB and 200 µL aliquots were loaded into wells of a 96-well polystyrene microtiter plate (BD Falcon, USA). To study the effect of the alkaloids on biofilm formation, PIP or RES (0.5 and 5.0 µg/mL) were added to each well. Polystyrene culture tubes were adopted as a second biofilm model, exhibiting a different oxygen/nutrient ratio and surface area. A 500 µL aliquot of the 1000-fold diluted culture, with or without PIP or RES, was added to the tubes; biofilms were allowed to develop in the tubes and plates at 37°C for 48 h under stationary conditions.

To study the effect of PIP and RES (0.5 and 5.0 µg/mL) on biofilm dispersal, 200 µL of the diluted overnight bacterial culture was loaded into wells of a 96 well plate and the plates were incubated at 37°C for 48 h under stationary conditions to allow biofilm formation. The planktonic cells were then discarded and the biofilms were washed twice with sterile phosphate buffered saline (PBS, pH 7.0) to remove non-adherent cells. PIP and RES (0.5 and 5.0 µg/mL) were added to each well with or without antibiotics ciprofloxacin (5 µg/mL) and azithromycin (15 µg/mL) and incubated for 24 h at 37°C. After the incubation period, the wells were washed twice with sterile PBS (pH 7.0) to remove non-adherent cells. The adhered biomass was quantified using the crystal violet assay [34]. Briefly, wells were gently washed twice with DI water to remove non-adherent, planktonic cells. After air drying (10 min), 200 µL of a 0.1% CV solution was added to each well (15 min). Plates were rinsed with DI water, air dried and then 200 µL of 95% ethanol was added to solubilize the CV stain bound to the surface-attached biofilms. The absorbance at OD_570_ (Tecan Infinite M200 Pro, Switzerland) was measured to estimate the biofilms that were formed. Biofilm levels (OD_570_) were normalized to the level of bacterial growth (OD_600_, determined separately) to decrease potential bias in the measurements introduced due to differences in growth rate.

### Quantitative Reverse Transcription PCR (qRT-PCR)

To detect the effect of PIP and RES on transcription of selected genes associated with motility and biofilm formation, overnight grown cells of *E. coli* CFT073 and the flagellar mutant (Δ*fli*C) were diluted 1000-fold in LB, mixed with PIP or RES (0.5 and 5.0 µg/mL) and incubated in 50 mL tubes at 37°C for 48 h. Bacteria were pelleted (5000×*g*, 10 min), and their total RNA was extracted using TRIzol (Invitrogen, CA) and purified using the Direct-zol RNA Miniprep kit (Zymo Research Corp., USA) following the manufacturers' instructions. RNA quality was confirmed by measuring the OD_260/280_ ratio using a microplate reader. Expression of genes associated with motility and biofilm formation (Table S1) was quantified using a two-step qRT-PCR analysis. The RNA concentration was calculated by measuring the absorbance at 260 nm and 300 ng of RNA was used for cDNA synthesis using the M-MLV Reverse Transcriptase Kit (Invitrogen). qRT-PCR was carried out in the ABI Prism 7900 HT thermal cycler (Applied Biosystems) using the Power SYBR Green PCR Master Mix (Applied Biosystems) under the following conditions: 50°C for 2 min, initial denaturation at 95°C for 10 min, and 45 cycles of 15 s at 95°C and 1 min at 60°C. Results were analyzed with SDS software (v. 2.2 Applied Biosystems). Data were normalized to the endogenous reference gene (*gap*A) and analyzed by the threshold cycle method (2^−ΔΔ*C*^ *_T_*) [35]. This experiment was repeated three times with independently isolated RNA samples.

### Penetration of antibiotics in biofilm

Biofilms of *E. coli* CFT073 were developed for 48 h on black, polycarbonate membranes according to the method of Singh et al. (2010) [24] with a few modifications (Figure S1). Briefly, 20 µL of an overnight culture of *E. coli* was seeded on black hydrophilic, polycarbonate membranes (Millipore, CA; 13 mm diameter; 0.4 µm pore size) placed on LB agar plates. The plates were incubated at 37°C for 48 h to allow growth of biofilms on the membranes. The membrane-supported biofilms were transferred onto fresh LB-agar plates at 24 h and 48 h to avoid decrease in biofilm growth due to depletion of nutrients in the medium. After 48 h, the biofilms formed on the membranes were washed by dipping the membranes in sterile PBS to remove the non-adherent cells. A 100 µL drop of *E. coli* CFT073 (OD_600_ = 0.5) was spread on Mueller Hinton agar (MHA) plates; the membrane-supported biofilms were transferred to this plate and covered with a sterile nitrocellulose membrane (13 mm; pore size, 0.4 µm). An antibiotic disc (azithromycin, ATH 15 µg; ciprofloxacin, CIP 5 µg; Alere Inc., Canada) pre-moistened with 20 µL of sterile DI water (to prevent antibiotic movement through the biofilm via capillary action) was placed on the nitrocellulose membranes. Drops of PIP or RES were added to the antibiotic discs (10 µL, 0.5 and 5.0 µg/mL). The experimental setup for penetration of antibiotics in *E. coli* CFT073 is shown in Figure S1. Blank assemblies had the same configuration with one difference: the polycarbonate membranes did not contain biofilms. The plates were incubated for 24 h at 37°C, after which the zone of growth inhibition on MHA plates was measured and recorded. Experiments were performed in triplicate.

### Microscopy

Confocal laser scanning microscopy (CLSM) was used to image the biofilms formed on polycarbonate membranes. The cells were stained with SYTO9 (Molecular Probes, Invitrogen). Subsequently, z-stack images of stained biofilms were captured using a Zeiss CLSM system (Jena, Germany) using a 488-nm argon laser. Three independent biofilm samples were imaged at 5 frames each and used to calculate the average thickness of the biofilms.

### Statistical analysis

Data was analyzed by 2-tailed Student's *t*-test with Bonferroni post-hoc analysis to compare replicate means. Differences with *p-*values <0.05 were considered significant. The t-statistic value was calculated to confirm normality of the data distribution.

## Results

### Effect of PIP and RES on bacterial growth and biofilm formation

**Figure 2:**
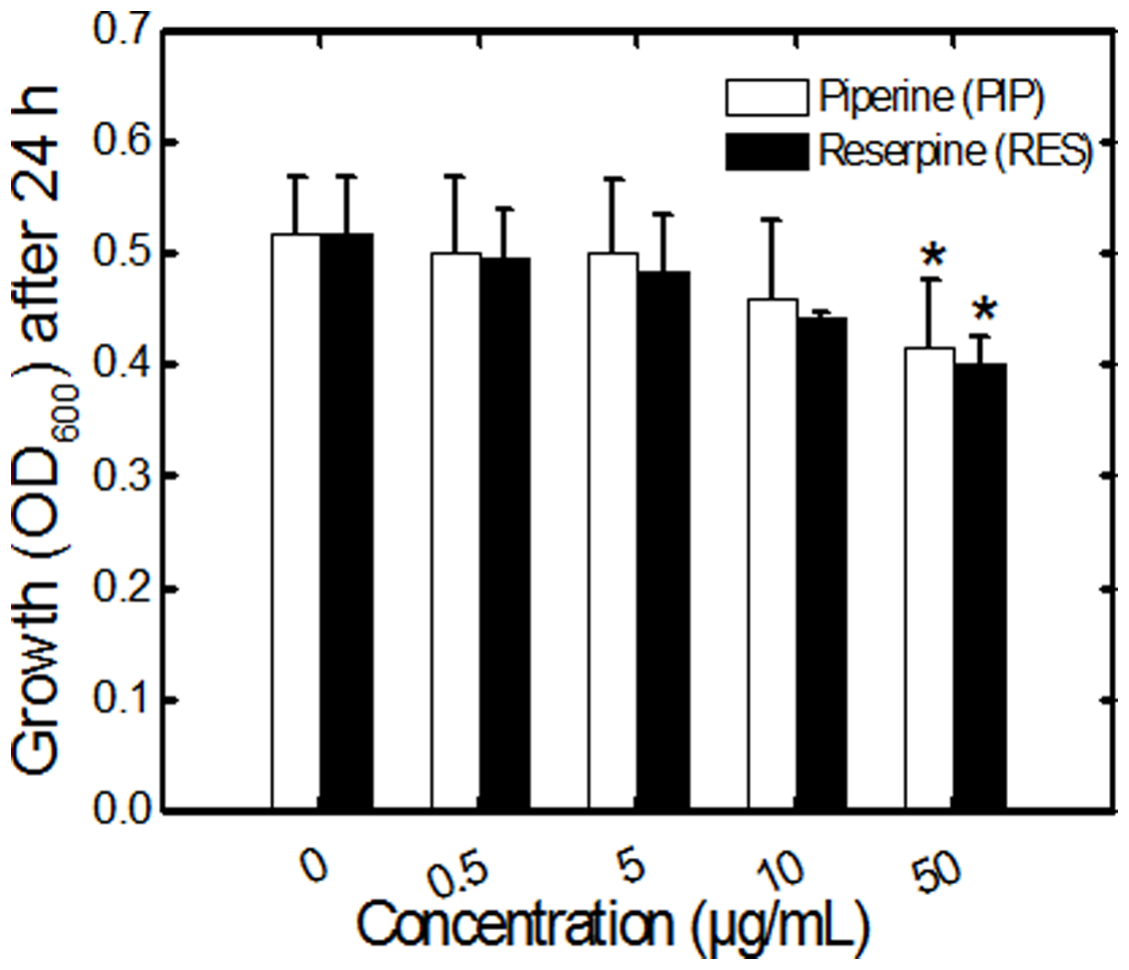
Growth levels of *E. coli* CFT073 in LB medium after 24 h. Bacterial growth was estimated as OD_600_ in the presence and absence of PIP and RES in 96 well plates. Values shown denote the mean + SD for three experiments with triplicate wells per experiment. The 24 h growth curves are presented in Figure S2.

**Figure 3:**
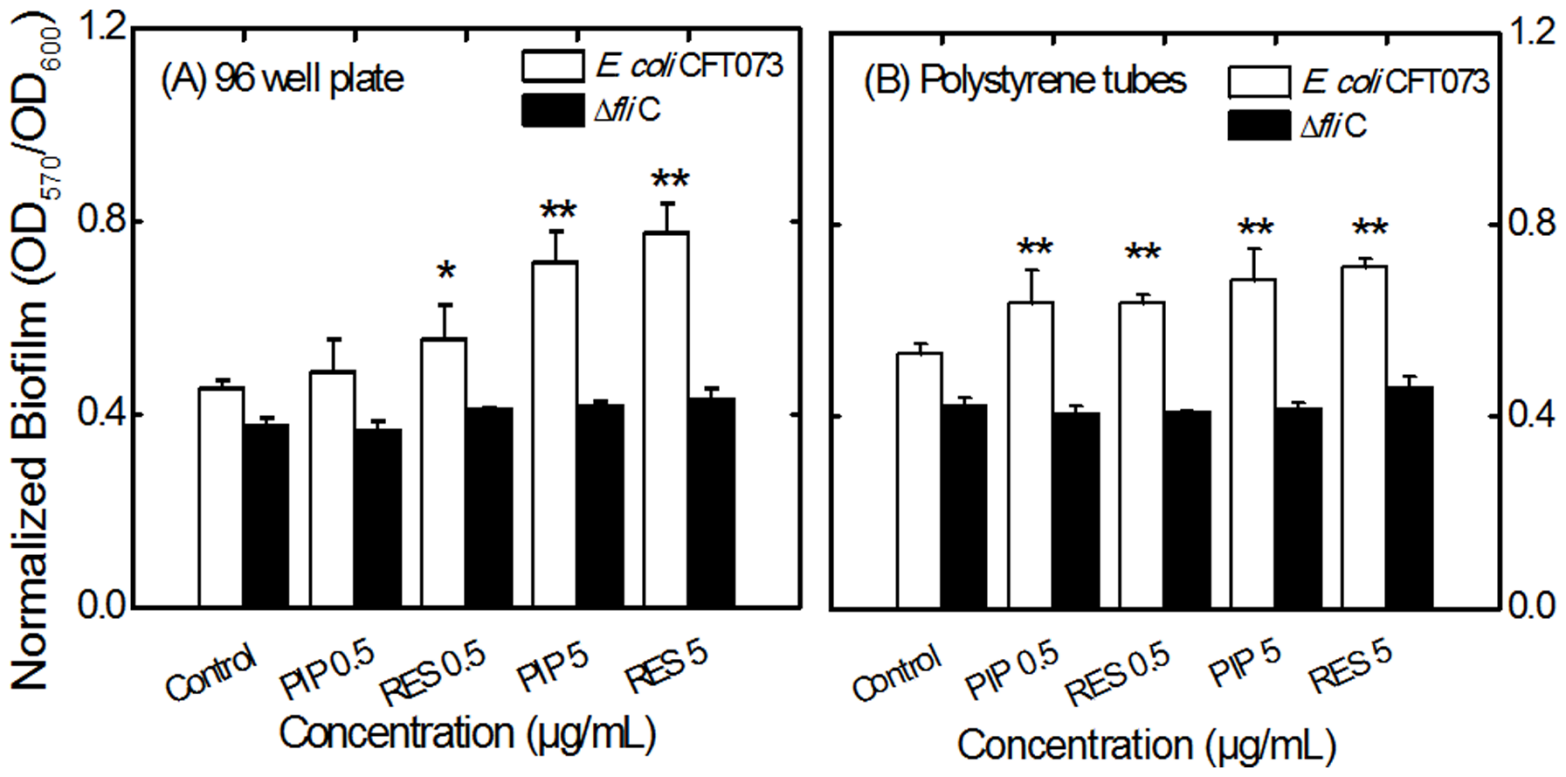
Effect of PIP and RES on 48 h biofilm levels of *E. coli* CFT073 (white bars) and the flagellar mutant *E. coli* Δ*fli*C (black bars) in (A) 96 well plates and (B) polystyrene tubes. The biofilm values (OD_570_) were normalized with respect to growth (OD_600_). Abbreviations: PIP 0.5 or RES 0.5, piperine or reserpine at 0.5 µg/mL (*e.g.*, PIP 0.5 indicates piperine at 0.5 µg/mL). Values shown denote the mean + SD for three experiments. * and ** indicate statistically significant differences with respect to the control with values *p*<0.05 and *p*<0.01, respectively.

*E. coli* CFT073 was grown in liquid LB medium in the presence and absence of PIP and RES. Higher concentrations of these alkaloids (50 µg/mL) slightly decreased the growth of *E. coli* (Figure 2, Figure S2). A similar trend was observed for *E. coli* Δ*fli*C mutant (data not shown). Concentrations of the alkaloids 0.5 and 5 µg/mL were chosen for subsequent motility and biofilm experiments since they had negligible effect on the growth of *E. coli* CFT073. Biofilm values (OD_570_) were normalized by growth levels (OD_600_) to compensate for differences in the growth rate as described in the literature [36], [37]. The normalized biofilm levels are presented in Figure 3 for biofilms pre-treated with alkaloids, and in Figure S3 for biofilms post-treated with alkaloids. Non-normalized biofilm data are presented in Figure S4 for reference. In the case of biofilm pre-treatment, *E. coli* CFT073 formed more biofilm when incubated with PIP or RES. Biofilm formation increased by 36% compared to the control for 5 µg/mL PIP in 96 well plates (Figure 3A). RES resulted in increased biofilm formation of 19% and 41% at 0.5 and 5 µg/mL, respectively. A similar trend was observed for biofilms formed in polystyrene tubes (Figure 3B). Interestingly, the presence of PIP or RES had no effect on the level of biofilm formation for the *E. coli* flagellar mutant (*E. coli* Δ*fli*C) suggesting that the effect of PIP and RES on *E. coli* biofilms is dependent on the knocked-out flagellar gene (*fli*C) and/or on flagellar function. Furthermore, when the alkaloids were added to a fully developed biofilm (post-treatment), the biofilm levels did not change significantly for the flagellar mutant (*fli*C) after 24 h incubation (Figure S3). However, with the wild type *E. coli* CFT073, biofilms (post-treated) increased by 11% and 16% compared to the control (untreated biofilm) for 0.5 and 5 µg/mL PIP and 14% and 22% for 0.5 and 5 µg/mL RES in 96 well plates (Figure S3A).

### Effect of PIP and RES on bacterial motility

**Figure 4:**
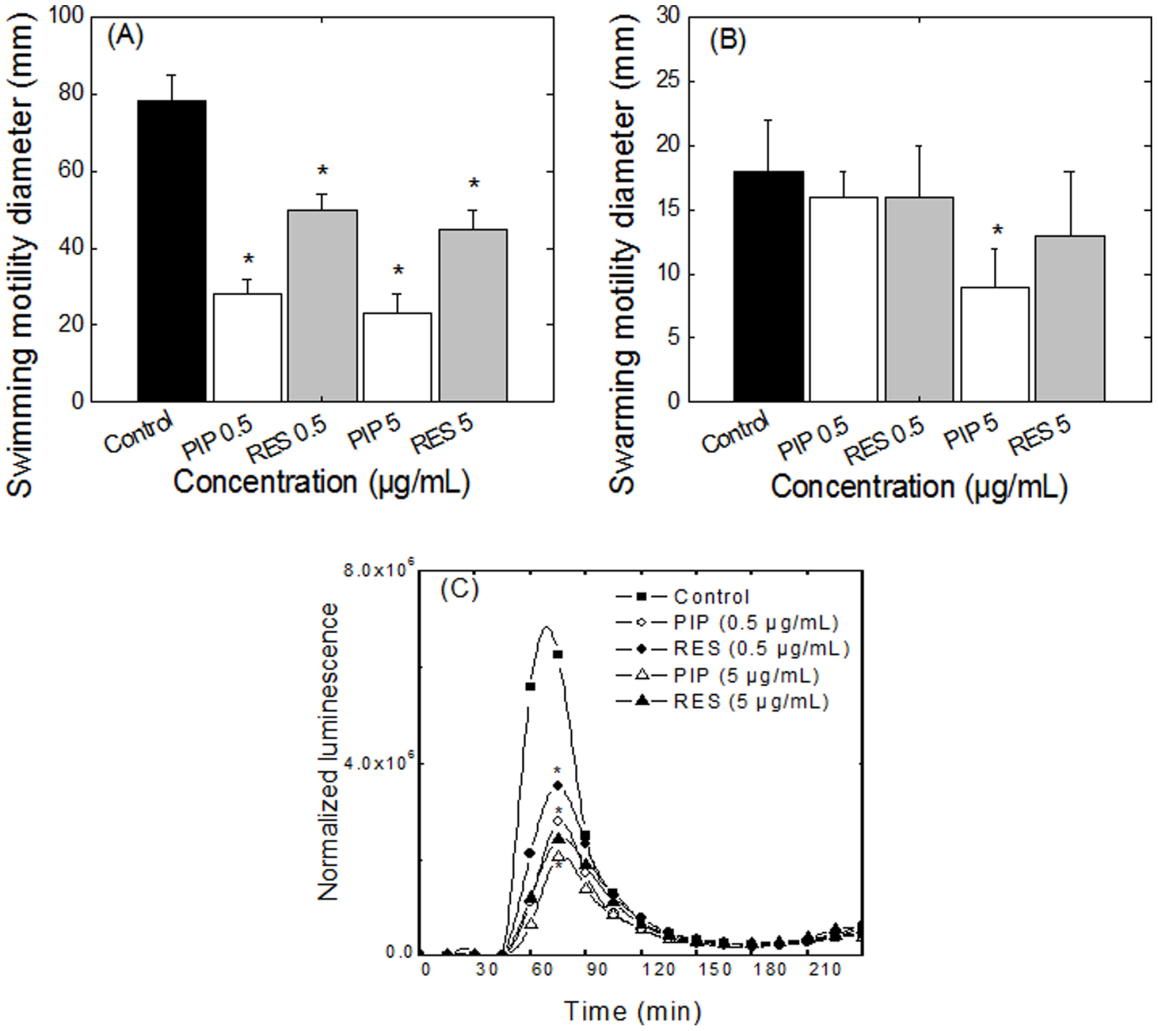
Effects of PIP and RES on *E. coli* CFT073 motility and flagellin expression. (A) swimming and (B) swarming motility of *E. coli* CFT073, (C) expression of flagellin in *E. coli* P*fli*C-lux in presence and absence of PIP and RES. * indicates statistically significant difference in values (*p*<0.05) with respect to the control.

Flagellar function was assessed in the presence and absence of PIP and RES by quantifying bacterial swimming and swarming motilities. *E. coli* swimming motility was drastically affected even at 0.5 µg/mL of PIP (Figure 4A). Concentrations of 0.5 and 5.0 µg/mL PIP decreased swimming motility in *E. coli* by 64% and 71%, respectively, compared to the control. RES also reduced the swimming motility by 36% and 42% at 0.5 and 5 µg/mL, respectively. Interestingly, a 10-fold increase in PIP or RES concentration only decreased the swimming diameter by a few percent, suggesting possible saturation of the effect at low alkaloid concentrations. *E. coli* CFT073 exhibited a swarming diameter of 18±4 mm in the absence of PIP or RES (Figure 4B). Addition of PIP at a concentration of 5 µg/mL decreased the swarming diameter of *E. coli* by 50%. RES had no significant effect on swarming motility of *E. coli*.

### Downregulation of fliC in presence of PIP and RES during early stages of growth

The effect of PIP and RES on expression of the flagellin gene, *fliC*, was quantified by monitoring the flagellin bioluminescent reporter strain of *E. coli* P*_fli_*_C_–lux during early stages of growth (Figure 4C). A control experiment conducted with the Steady-Glo assay confirmed that neither PIP nor RES interfered with the luminescence measurements at the concentrations tested (Figure S5A and B). A change in the transcription of *fli*C in the *fli*C*-lux* reporter correlates with a change in the luminescence signal. A significant reduction in flagellin expression was observed for both PIP and RES (*p*<0.01, Figure 4C). At 5 µg/mL, PIP and RES resulted in 67% and 61% reduction in the luminescence signal, respectively, compared to the 55% and 43% reduction at 0.5 µg/mL.

### Expression of genes associated with motility and adhesion at stationary phase

The transcription of genes involved in bacterial colonization was quantified after 48 h of exposure of *E. coli* CFT073 to PIP and RES. It is noteworthy that the qRT-PCR results present a snapshot of status of planktonic cells in stationary phase after 48 h of static growth. Planktonic cells were deliberately chosen for gene expression studies since cells in a biofilm are known to have higher expression of adhesion genes and lower expression of motility genes. Therefore, potential effect of alkaloids on gene expression could be masked by the already altered gene expression for cells in a biofilm. Stationary phase planktonic cells incubated with 5 µg/mL of PIP exhibited a reduced expression of *fli*C (Table 1). Genes expressing the major flagellar motor proteins (*motA* and *motB*) were drastically downregulated for both concentrations of PIP and RES tested. Furthermore, expression of the fimbriae-associated genes exhibited a dose-dependent increase compared to the control (Table 1). Expression of *fim*A, responsible for the production of the fimbrial subunit protein, increased 3.4 and 5.2 fold in the presence of 5 µg/mL of PIP and RES, respectively. Expression of *pap*A increased 1.2 and 2.5 fold in the presence of 5 µg/mL of PIP and RES, respectively. Expression of *uvr*Y also increased significantly with both alkaloids (2.7 and 2.4 fold increase for 5 µg/mL of PIP and RES, respectively). Overall, the qRT-PCR results suggest that the observed alkaloid-induced biofilm increase could potentially be a result of the increase in the expression of adhesion-associated genes (*fim*A, *uvr*Y and *pap*A) and the decrease in the expression of motility-associated genes (*fliC*, *motA and motB*).

**Table 1:** Expression of genes responsible for motility and biofilm formation in *E. coli* CFT073 wild type after 48 h of growth in presence and absence of PIP and RES.

	Piperine		Reserpine	
	0.5 µg/mL	5 µg/mL	0.5 µg/mL	5 µg/mL
*fliC*	0.90 (0.4)	**0.68 (0.19)**	0.74 (0.19)	0.91 (0.25)
*motA*	**4.1×10^−5^ (5×10^−2^)**	**0.030 (0.01)**	**8.4×10^−6^ (1×10^−3^)**	**1.2×10^−6^ (5×10^−3^)**
*motB*	**0.45 (0.09)**	**0.38 (0.05)**	**0.55 (0.19)**	**0.48 (0.19)**
*fimA*	**1.4 (0.03)**	**3.4 (0.17)**	**2.1 (0.26)**	**5.2 (0.35)**
*uvrY*	**1.6 (0.15)**	**2.7 (0.40)**	1.1 (0.30)	**2.4 (0.5)**
*papA*	1.1 (0.15)	**1.2 (0.17)**	0.89 (0.40)	**2.5 (0.35)**

Relative mRNA quantities were normalized to that of a housekeeping gene, *gap*A. Results represent mean fold change values ± SD (in parentheses) for three independent experiments with respect to control. Values in bold font indicate statistically significant differences in mRNA relative value with respect to the control (*p*<0.05).

### Penetration of antibiotics through E. coli biofilm and biofilm dispersal

**Figure 5:**
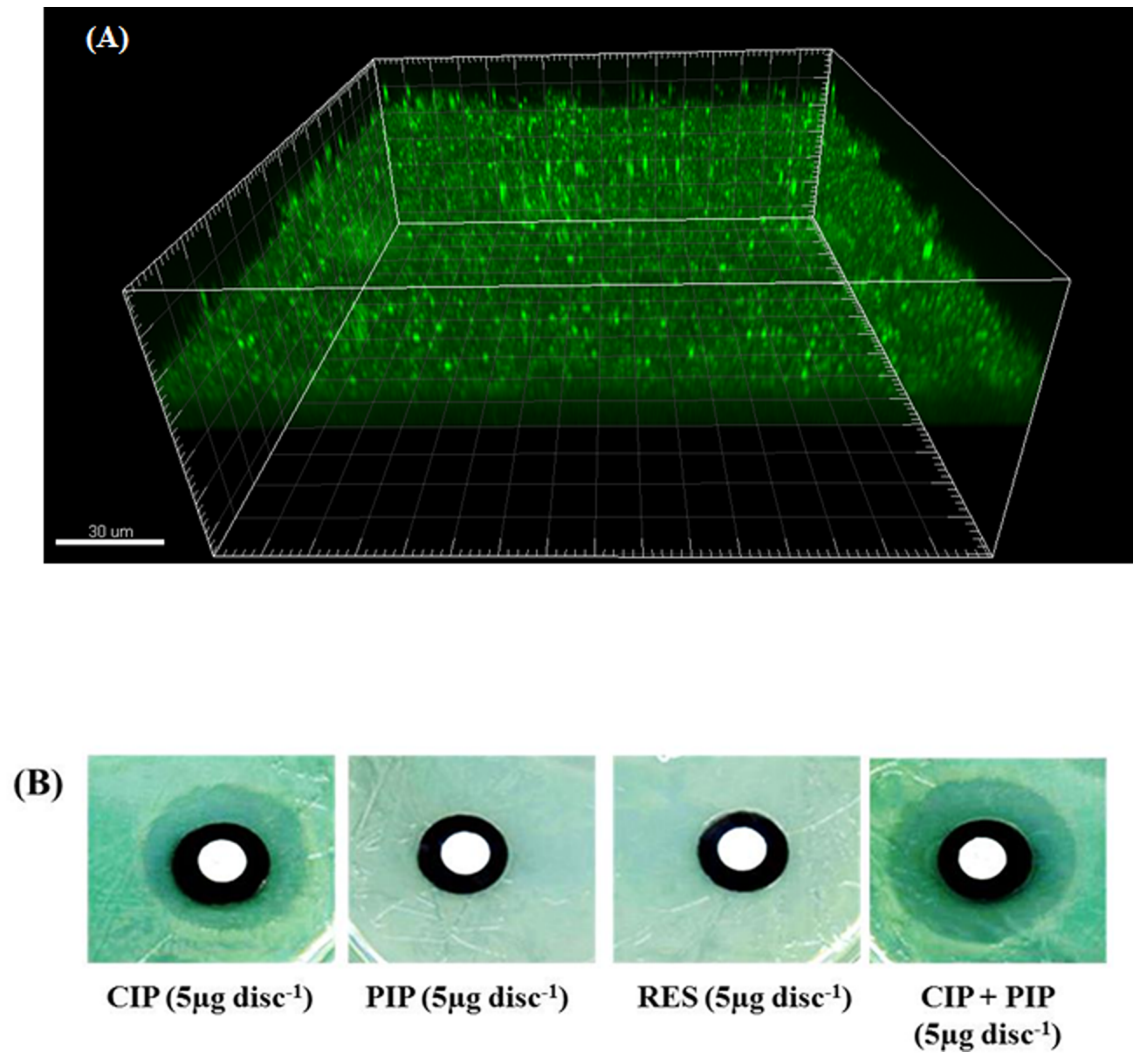
Effects of PIP and RES on antibiotic penetration through biofilms. (A) Confocal image of 48 h *E. coli* CFT073 biofilm formed on polycarbonate membranes, 3-dimensional reconstruction. Cells were stained with the fluorescent SYTO9 dye and are shown in green. (B) Representative images of zones of inhibition due to different treatments.

Biofilms of *E. coli* were developed on polycarbonate membranes for 48 h and had a uniform thickness of 14.2 (±3) µm (Figure 5A). The presence of *E. coli* biofilms on the membranes decreased the penetration of antibiotics (5 µg CIP and 15 µg ATH) through the membrane compared to discs without a biofilm, as indicated by the decreased zone of inhibition around the discs with biofilm compared to that without biofilm (Table 2). Figure 5B shows representative images of inhibition zones around the membrane assembly containing the biofilms. The presence of PIP increased the penetration of antibiotics through the biofilm as indicated by the larger inhibition zone around the discs compared to the control biofilm without the alkaloid (Table 2, Figure 5B). RES had no significant effect on the zone of inhibition for either of the two antibiotics (Table 2). PIP and RES alone (5 µg/mL) had no inhibitory effect on *E. coli* CFT073 and showed no zone of inhibition when applied to a membrane without antibiotics.

**Table 2:** Penetration of antibiotics ciprofloxacin (CIP) and azithromycin (ATH) through pre-formed *E. coli* CFT073 biofilms in presence and absence of piperine (PIP) and reserpine (RES).

Compounds	Zone diameter (mm)	
**Antibiotics**	**Without biofilm**	**With biofilm**
CIP (5 µg/disc)	30±1	24±2
ATH (15 µg/disc)	24±1	18±2
**Alkaloids**		
PIP (5 µg)	0	0
RES (5 µg)	0	0
**Combination**		
CIP + PIP	33±2	28±2*
CIP + RES	31±2	25±1
ATH + PIP	27±2	24±1*
ATH + RES	24±2	19±1

* Indicates statistically different values (p<0.05) when compared with antibiotic alone.

**Figure 6:**
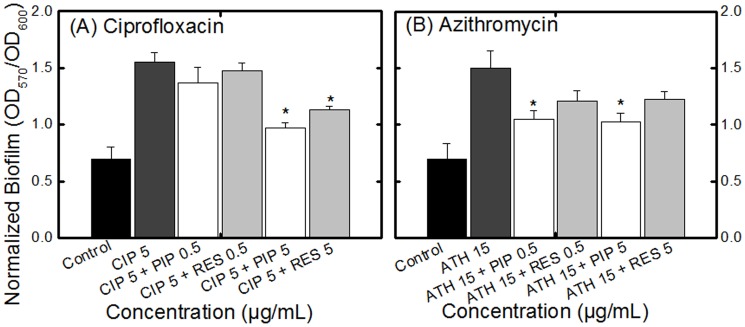
Effect of PIP and RES on the efficacy of antibiotics (A) ciprofloxacin (CIP 5 µg/mL) and (B) azithromycin (ATH 15 µg/mL) towards fully developed *E. coli* CFT073 biofilms. The biofilms were allowed to form for 48 h and were subsequently incubated with the alkaloids for 24 h. Control indicates biofilms not treated with antibiotic or alkaloids. All biofilm values (OD_570_) are normalized with growth (OD_600_). Values for OD_570_ are presented in Figure S6. Abbreviations: PIP 0.5 or RES 0.5, piperine or reserpine at 0.5 µg/mL (e.g., PIP 0.5 indicates piperine at 0.5 µg/mL). * indicates statistically significant (*p*<0.05) decrease in biofilm level for alkaloid + antibiotic treatment compared to the respective antibiotic treatment (CIP 5 or ATH 15).

To assess the effect of PIP and RES on the ability of antibiotics to disperse a fully developed biofilm, antibiotics (same dosage used for antibiotic penetration assay) were added to a 48 h biofilm, pre-formed in a 96 well microtiter plate. The overall growth level significantly decreased after 24 h of incubation with antibiotics but the ratio of sessile to planktonic cells increased significantly for biofilms treated with CIP (Figure 6A) or ATH (Figure 6B). Adding PIP or RES (at 5 µg/mL) along with the antibiotic, however, significantly increased the ability of both antibiotics to destroy the biofilm compared with CIP or ATH alone, as manifested by the decrease in the ratio of sessile to planktonic cells (with the exception of ATH+RES). Non-normalized biofilm data are presented in Figure S6. It is noteworthy that many of the trends visible in Figure 6 are not obvious in Figure S6 which highlights the importance of normalizing biofilm data.

## Discussion

### Preference of bacteria to adopt a sessile phenotype

We investigated two natural compounds, PIP and RES, for their ability to affect the motility and biofilm formation of uropathogenic *E. coli* CFT073. The alkaloids exhibited minimal effect on bacterial growth; however, biofilm formation significantly increased for bacterial cells incubated with both PIP and RES. These results are in agreement with earlier reports wherein cranberry and pomegranate materials, also containing alkaloids, were observed to block bacterial motility [9], [10] and increase biofilm formation [11]. Although at first instance, increase in biofilm formation seems a disadvantage in a therapeutic context, it should be noted that this effect is accompanied by a significant decrease in motility which has been reported to decrease the spread of infection [3], [38]. *E. coli* possess peritrichous flagella (5–10 flagella distributed around the cell) which contribute to bacterial swimming in liquid media and, to some extent, to their movement along surfaces, thereby promoting the spread of infection [39]. We report a decrease in bacterial motility (confirmed by a decrease in the expression of motility associated genes) which can potentially hamper the dissemination of infection.

It is noteworthy that PIP decreased both swimming and swarming motilities. Unlike swimming motility which is a single-cell act, swarming motility is a social behaviour and bacterial cells have to communicate to be able to swarm [40]. This communication, known as quorum sensing, is a major virulence factor and decreased swarming has been correlated with decreased quorum sensing, and thus, decreased virulence [40]. Thus, it would be interesting to evaluate in future studies whether PIP could act as an anti-quorum sensing agent.

Another interesting observation was the inability of PIP and RES to affect biofilm formation for the flagellar mutant strain (Δ*fli*C). The flagellar mutant exhibited lower biofilm levels compared to the wild type (Figure 3A and 3B), as reported previously [25], [41], [42]. The level of biofilm formation for the flagellar mutant stayed the same in the presence of PIP and RES, while biofilm levels for the wild type strain increased significantly. This could suggest that PIP and RES affect UPEC biofilm formation mainly through interaction with bacterial flagellar protein FliC, or the associated gene. This theory is reinforced by the fact that both alkaloids resulted in a decrease in bacterial motility (which is heavily dependent on flagellar function) and a decreased expression of flagellar genes (*fliC, motA* and *motB*). There is no report in the literature describing how or why alkaloids might result in a decreased expression of flagella-associated genes. It has been suggested that under stress conditions, shutting down the flagellar gene expression is one of the strategies cells adopt to avoid waste of energy for making new flagella [43]. Moreover, it has been reported that inactivation of genes responsible for flagella production also triggers expression of adhesion-associated genes [43], which is in accordance with our results.

### Enhanced antibiotic action towards biofilm

Formation of biofilms on urinary catheters significantly hampers the treatment of infection [44], [45]. Biofilms can decrease the access of antimicrobial agents to cells by a number of mechanisms, the most important of which is modulating the penetration of the drugs or degrading drug molecules [22]. Our results indicated that adding antibiotics does not fully disperse biofilms; it can even result in an increase in the ratio of cells that adopted a sessile lifestyle. This effect has been reported before for antibiotics [46] and other stressors [36] where stress caused the bacterial cells to adopt a biofilm lifestyle as a defensive mechanism. Interestingly, adding PIP and RES along with the antibiotics resulted in a decrease in the levels of biofilm, suggesting that the presence of alkaloids decreased the ability of the cells in the biofilm matrix to escape the inhibitory action of antibiotics. Antibiotic penetration studies indicated that PIP significantly increased the penetration of antibiotics through the *E. coli* biofilm which could partially explain the enhanced antibiotic action in the presence of this alkaloid. The alkaloids PIP and RES used in the present investigation are known bioavailability enhancers [47], [48]. Many studies have reported the physiological effects of black pepper, its extracts, or its major active component, PIP [47]–[50]. Furthermore, oral administration of *P. longum* (long pepper) containing PIP to poultry has been reported to enhance the therapeutic efficacy of the antibiotic oxytetracycline [51]. Our results support the reports in the literature and further present a possible mechanism for the therapeutic effects of PIP, namely enhancing the diffusion of antibiotics in bacterial biofilms.

## Conclusions

In conclusion, we have shown that sub-inhibitory concentrations of PIP or RES can reduce flagellin expression and bacterial motility in UPEC, potentially decreasing the dissemination of infection. Although addition of PIP or RES resulted in an increase in biofilm formation, these alkaloids were observed to also increase the ability of antibiotics to destroy *E. coli* biofilms, suggesting a potentially useful therapeutic application for these compounds.

## Supporting Information

Figure S1 **Graphical representation of the experimental setup for penetration of antibiotics and alkaloids (PIP or RES) in pre-established** ***E. coli*** **CFT073 biofilms.**
(TIF)

Figure S2 **Growth curves for** ***E. coli*** **CFT073 in 96 well plates in LB medium.** Growth of the bacterium was quantified as OD_600_ in presence and absence of (A) PIP and (B) RES. Values shown denote the mean of three experiments in triplicate wells per experiment.
(TIF)

Figure S3 **Effect of PIP and RES on fully developed (A) normalized and (B) non-normalized (B) biofilms of** ***E. coli*** **CFT073 (white bars) and the flagellar mutant** ***E. coli*** **Δ** ***fli*** **C (black bars).** Abbreviations: PIP 0.5 or RES 0.5, piperine or reserpine at 0.5 µg/mL (e.g., PIP 0.5 indicates piperine at 0.5 µg/mL). The biofilms were allowed to form for 48 h and were subsequently incubated with the alkaloids (PIP or RES) for 24 h and the biofilm values (OD_570_) were normalized with growth (OD_600_). Values shown denote the mean + SD from three experiments and * indicates statistically significant difference in values with *p*<0.05 with respect to the control.
(TIF)

Figure S4 **Effect of PIP and RES on 48 h biofilm levels of** ***E. coli*** **CFT073 (white bars) and the flagellar mutant** ***E. coli*** **Δ** ***fli*** **C (black bars) in (A) 96 well plates and (B) polystyrene tubes.** Abbreviations: PIP 0.5 or RES 0.5 indicates piperine or reserpine at 0.5 µg/mL and PIP 5 or RES 5 indicates 5 µg/mL, respectively. The biofilms were allowed to form for 48 h in presence or absence of the alkaloids (piperine or reserpine). Values shown denote the mean + SD from three experiments and * indicates statistically significant difference in values with *p*<0.05 with respect to the control.
(TIF)

Figure S5 **Interference of alkaloids with luminescence assay.** Potential interference of (A) PIP and (B) RES with luminescence was analysed using Steady Glo assay kit. PIP or RES (0.5, 5 and 50 µg/mL) were added to the luciferase assay system in 96 well plates. Luminescence was recorded at 15 min intervals for up to 5 h to determine the effect of PIP or RES on luminescence. Interference with luminescence was not observed at any of the concentrations of PIP and RES tested. PIP or RES alone without luciferase showed a luminescence reading of zero (data not shown).
(TIF)

Figure S6 **Effect of PIP and RES on the efficacy of antibiotics (A) ciprofloxacin (CIP 5 µg/mL) and (B) azithromycin (ATH 15 µg/mL) towards fully developed biofilms of** ***E. coli*** **CFT073.** The biofilms were allowed to form for 48 h and were subsequently incubated with the alkaloids for 24 h. Control indicates biofilm not treated with antibiotics or alkaloids. Values presented are non-normalized. Abbreviations: PIP 0.5 or RES 0.5, piperine or reserpine at 0.5 µg/mL (e.g., PIP 0.5 indicates piperine at 0.5 µg/mL). * indicates statistically significant (*p*<0.05) decrease in biofilm level for alkaloid + antibiotic treatment compared to the respective antibiotic treatment (control).
(TIF)

Table S1 ***E. coli*** **strains, plasmids and primers used in the study.**
(DOCX)
