# Supplementary figures and images for: Alkaloids Modulate Motility, Biofilm Formation and Antibiotic Susceptibility of Uropathogenic Escherichia coli
Source: PLoS One. 2014 Nov 12;9(11):e112093. doi: 10.1371/journal.pone.0112093 (PMC4229180; doi:10.1371/journal.pone.0112093)

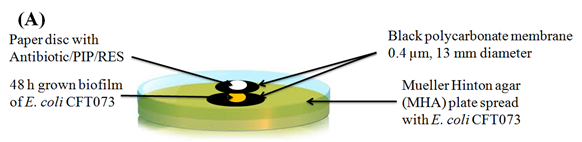

Supplement: Figure S1 — Graphical representation of the experimental setup for penetration of antibiotics and alkaloids (PIP or RES) in pre-established E. coli CFT073 biofilms. (TIF) [file pone.0112093.s001.tif]

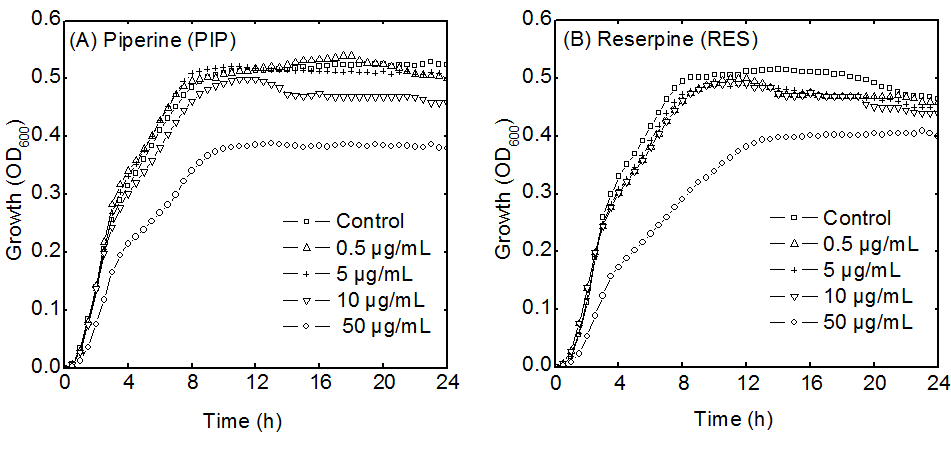

Supplement: Figure S2 — Growth curves for E. coli CFT073 in 96 well plates in LB medium. Growth of the bacterium was quantified as OD600 in presence and absence of (A) PIP and (B) RES. Values shown denote the mean of three experiments in triplicate wells per experiment. (TIF) [file pone.0112093.s002.tif]

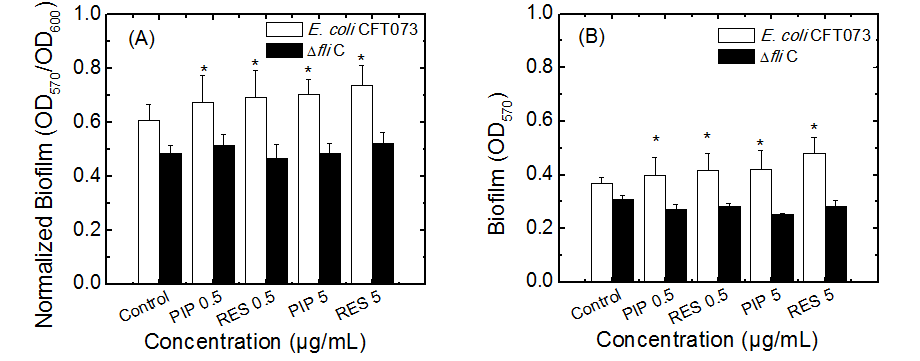

Supplement: Figure S3 — Effect of PIP and RES on fully developed (A) normalized and (B) non-normalized (B) biofilms of E. coli CFT073 (white bars) and the flagellar mutant E. coli Δ fli C (black bars). Abbreviations: PIP 0.5 or RES 0.5, piperine or reserpine at 0.5 µg/mL (e.g., PIP 0.5 indicates piperine at 0.5 µg/mL). The biofilms were allowed to form for 48 h and were subsequently incubated with the alkaloids (PIP or RES) for 24 h and the biofilm values (OD570) were normalized with growth (OD600). Values shown denote the mean + SD from three experiments and * indicates statistically significant difference in values with p<0.05 with respect to the control. (TIF) [file pone.0112093.s003.tif]

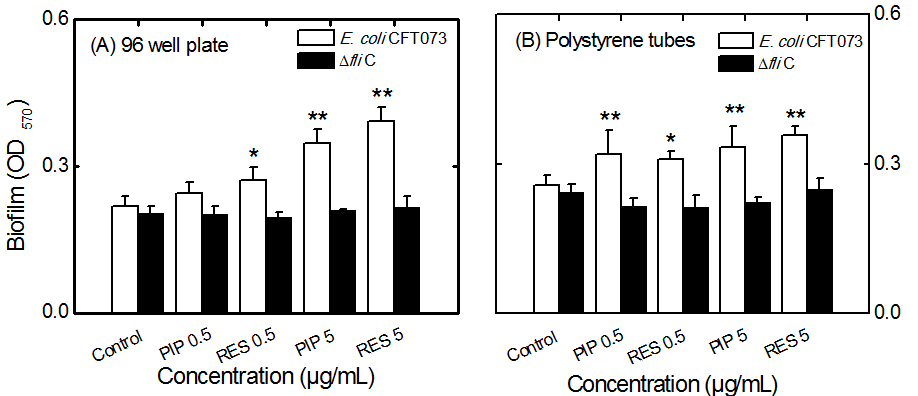

Supplement: Figure S4 — Effect of PIP and RES on 48 h biofilm levels of E. coli CFT073 (white bars) and the flagellar mutant E. coli Δ fli C (black bars) in (A) 96 well plates and (B) polystyrene tubes. Abbreviations: PIP 0.5 or RES 0.5 indicates piperine or reserpine at 0.5 µg/mL and PIP 5 or RES 5 indicates 5 µg/mL, respectively. The biofilms were allowed to form for 48 h in presence or absence of the alkaloids (piperine or reserpine). Values shown denote the mean + SD from three experiments and * indicates statistically significant difference in values with p<0.05 with respect to the control. (TIF) [file pone.0112093.s004.tif]

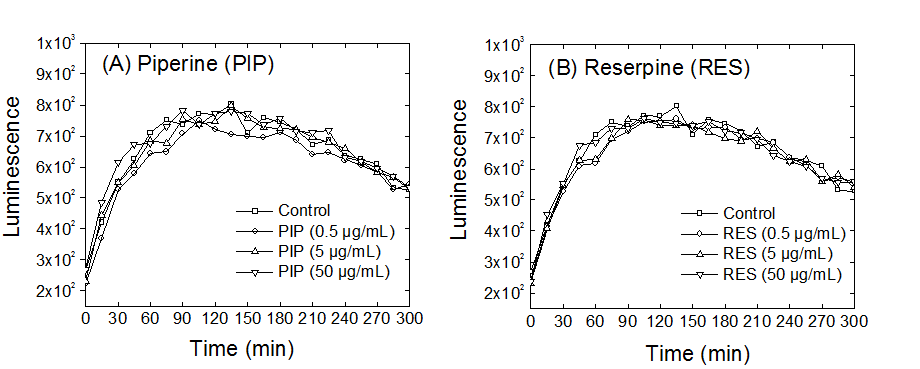

Supplement: Figure S5 — Interference of alkaloids with luminescence assay. Potential interference of (A) PIP and (B) RES with luminescence was analysed using Steady Glo assay kit. PIP or RES (0.5, 5 and 50 µg/mL) were added to the luciferase assay system in 96 well plates. Luminescence was recorded at 15 min intervals for up to 5 h to determine the effect of PIP or RES on luminescence. Interference with luminescence was not observed at any of the concentrations of PIP and RES tested. PIP or RES alone without luciferase showed a luminescence reading of zero (data not shown). (TIF) [file pone.0112093.s005.tif]

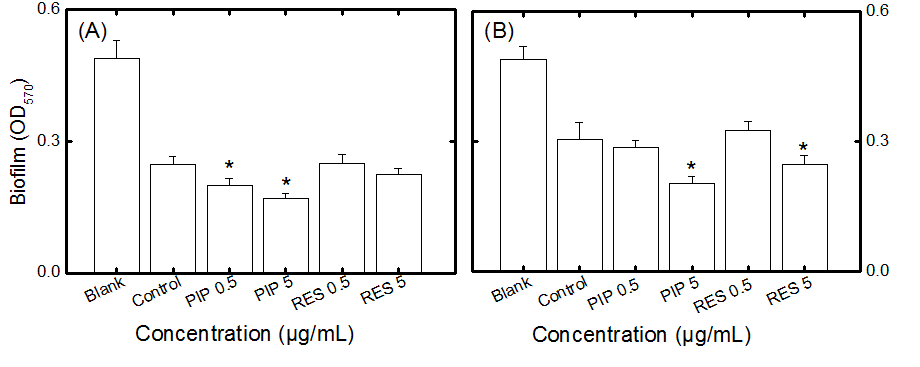

Supplement: Figure S6 — Effect of PIP and RES on the efficacy of antibiotics (A) ciprofloxacin (CIP 5 µg/mL) and (B) azithromycin (ATH 15 µg/mL) towards fully developed biofilms of E. coli CFT073. The biofilms were allowed to form for 48 h and were subsequently incubated with the alkaloids for 24 h. Control indicates biofilm not treated with antibiotics or alkaloids. Values presented are non-normalized. Abbreviations: PIP 0.5 or RES 0.5, piperine or reserpine at 0.5 µg/mL (e.g., PIP 0.5 indicates piperine at 0.5 µg/mL). * indicates statistically significant (p<0.05) decrease in biofilm level for alkaloid + antibiotic treatment compared to the respective antibiotic treatment (control). (TIF) [file pone.0112093.s006.tif]
