# Supplementary material for: Alkaloids Modulate Motility, Biofilm Formation and Antibiotic Susceptibility of Uropathogenic Escherichia coli
Source: PLoS One. 2014 Nov 12;9(11):e112093. doi: 10.1371/journal.pone.0112093 (PMC4229180; doi:10.1371/journal.pone.0112093)
Supplement: Table S1 — E. coli strains, plasmids and primers used in the study. (DOCX) [file pone.0112093.s007.docx]

**Table S1: *E. coli* strains, plasmids and primers used in the study.**

amp^r^ and kan^r^ are the ampicillin and kanamycin resistance, respectively.
